# Phenethyl Acetate as an Agonist of Insect Odorant Receptor Co-Receptor (Orco): Molecular Mechanisms and Functional Insights

**DOI:** 10.3390/ijms26114970

**Published:** 2025-05-22

**Authors:** Myungmi Moon, Jihwon Yun, Minsu Pyeon, Jeongyeon Yun, Jaehui Yang, Hye Duck Yeom, Geonu Lee, Yong-Seok Choi, Jaehyeong Lee, Junho H. Lee

**Affiliations:** 1 Department of Biotechnology and Department of Integrative Food, Bioscience and Biotechnology (BK21 FOUR), Chonnam National University, Gwangju 61186, Republic of Korea; audal12319@gmail.com (M.M.); dbswlgnjs128@gmail.com (J.Y.); vusalstn@naver.com (M.P.); curry204@naver.com (J.Y.); yangjh1219@naver.com (J.Y.); 2 GoPath Laboratories, Buffalo Grove, IL 60089, USA; hyeom@gopath.com; 3 Bioenvironmental Division, Chungnam Agricultural Research and Extension Services, Yesan 32418, Republic of Korea; lgw992@korea.kr (G.L.); yschoi92@korea.kr (Y.-S.C.); 4 Organic Agriculture Division, National Institute of Agricultural Sciences, Wanju 55365, Republic of Korea

**Keywords:** phenethyl acetate, odorant receptors (Ors), pest management strategies, odorant receptor co-receptor (Orco), two-electrode voltage clamp (TEVC), insect behavior

## Abstract

The insect olfactory system is vital for survival, enabling the recognition and discrimination of a wide range of odorants present in the environment. This process is mediated by odorant receptors (Ors) and the highly conserved co-receptor Orco. Insect Ors are structurally distinct from mammalian olfactory receptors, a divergence that presents unique advantages for developing insect-specific pest control strategies. In this study, we explored the molecular-level interactions between insect Ors and volatile organic compounds. Specifically, we investigated the response of Ors/Orco to phenethyl acetate (PA), a volatile compound found in the culture media of acetic acid bacteria. PA elicited activation in a concentration-dependent, reversible, and voltage-independent manner in Or1a, Or24a, and Or35a when combined with Orco, as well as in Orco homomers. Through molecular docking studies, we determined that the PA-binding site is localized to the Orco subunit, a highly conserved protein across diverse insect taxa. To further elucidate the role of key residues in the Orco homotetramer receptor, we performed site-directed mutagenesis. A mutational analysis identified W146 and E153 as critical residues for PA binding and activation. A double-mutant Orco receptor (W146A + E153A) exhibited a significant reduction in PA-induced activation compared to the wild-type receptor. These findings indicate that PA functions as an agonist for the *Drosophila melanogaster* Orco receptor and highlight its potential applications in chemosensory research and insect pest management strategies.

## 1. Introduction

**Figure 1:**
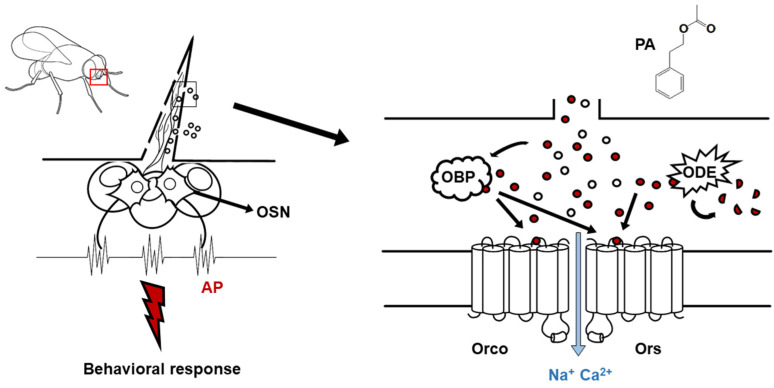
Schematic representation of the signal transduction pathway of insect odorant receptors (Ors). Olfactory sensory neuron, OSN; odorant-binding protein, OBP; odorant receptor co-receptor, Orco; action potential, Ap; odorant-degrading enzyme, ODE.

The insect olfactory system is essential for survival, playing a crucial role in locating food sources, identifying suitable oviposition sites, and recognizing potential mates [1,2,3]. This process is mediated by odorant receptors (Ors), a diverse family of proteins that enable insects to detect and discriminate among thousands of volatile compounds in their environment [4,5]. Unlike mammalian G-protein-coupled olfactory receptors, insect Ors form heteromeric ion channels in combination with the highly conserved odorant receptor co-receptor (Orco), allowing direct ion influx upon odorant binding [6,7]. Given their distinct molecular architecture and functional properties, insect Ors represent promising targets for developing species-specific pest control strategies, an area of increasing interest in agricultural and ecological research [8,9]. Despite advances in understanding the structural and functional mechanisms of insect Ors, key questions remain regarding their interactions with volatile organic compounds at the molecular level. One such compound is phenethyl acetate (PA), an aromatic ester widely found in the fermentation byproducts of acetic acid bacteria and in various fruits such as apples, bananas, and pears [10,11]. PA is a naturally occurring volatile organic compound (VOC) known for its characteristic floral and honey-like scent, making it a key component in the aroma profiles of many plant-derived substances. It plays an important ecological role as a semiochemical, mediating interspecies and intraspecies communication in various insect species. PA has been reported to function as a key kairomone for certain parasitoid wasps, facilitating host detection, and as a pheromone component in some insects, influencing mating and aggregation behaviors, as seen in in Figure 1 [12,13,14]. In addition to its role in insect–insect communication, PA has been implicated in olfactory-driven host-plant selection and feeding behaviors in herbivorous insects, including fruit flies and beetles. Recent studies suggest that PA can act as either an attractant or a repellent, depending on species-specific olfactory receptor (Or) tuning and neural processing mechanisms [14]. While its behavioral effects on insects have been documented, the molecular basis of PA perception remains poorly understood, particularly its interaction with Orco, the obligatory co-receptor of the insect odorant receptor complex. Despite the growing interest in PA as a bioactive volatile compound with potential applications in pest control, its precise mechanism of action on insect odorant receptors, particularly its role in modulating Orco-dependent ion channel activity, remains unclear. Addressing this knowledge gap is essential for developing targeted olfactory-based strategies for insect behavior manipulation, including the design of novel semiochemicals for species-specific pest management [15,16].

PA has been identified as a key volatile compound in host-plant selection for certain fruit flies and beetles. Additionally, it has been observed to play a role in pheromonal communication in some insects, influencing mating and aggregation behaviors [17,18]. While much of the existing research has focused on species-specific behavioral responses, the precise molecular interactions of PA with insect odorant receptors, particularly the Or/Orco complex, remain largely unexplored. This study aims to address this gap by investigating PA’s role in *D. melanogaster* and its potential functional relevance across insect taxa.

In this study, we investigate the molecular interaction between PA and insect Or/Orco complexes, using *D. melanogaster* as a model system. Through electrophysiological assays and molecular docking studies, we identify PA as an agonist of Orco and pinpoint key residues involved in ligand binding. PA was chosen because it is a volatile compound found in the fermentation byproducts of acetic acid bacteria and is known to influence insect olfactory behavior. Octanol, on the other hand, is a well-characterized ligand for *D. melanogaster* Ors and serves as a reference compound for comparative analysis. Additionally, we perform site-directed mutagenesis to determine how specific amino acid substitutions affect PA-induced receptor activation. By elucidating the molecular basis of PA–Orco interactions, our findings provide new insights into insect olfactory signal transduction and contribute to the broader goal of designing Or-targeted compounds for pest management applications.

## 2. Results

### 2.1. PA Activates Or1a, Or24a, and Or35a Receptors in Combination with Orco

**Figure 2:**
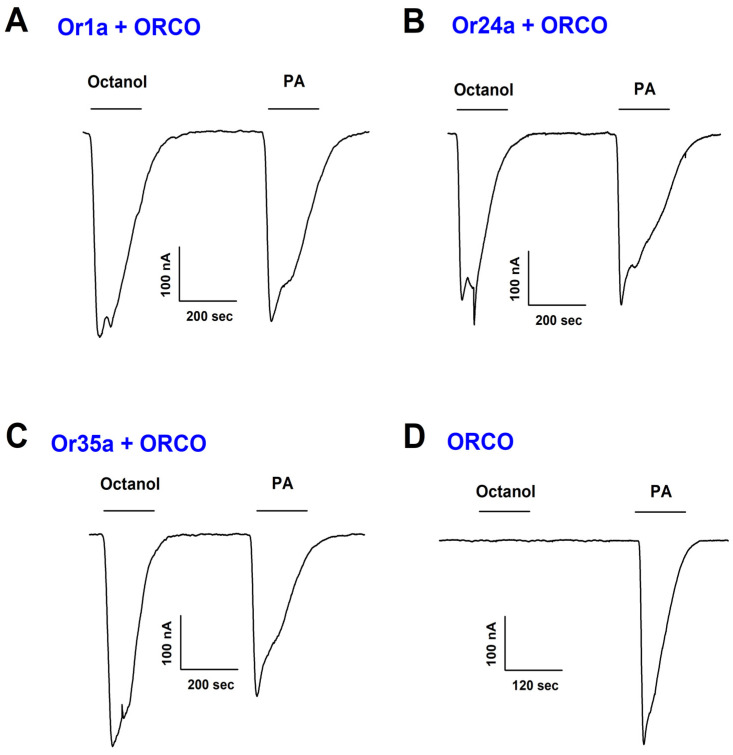
Activation of Or1a, Or24a, or Or35a co-expressed with Orco or Orco homotetramer by phenethyl acetate. A comparative electrophysiological analysis was performed to assess the activation of Or1a/Orco (**A**), Or24a/Orco (**B**), Or35a/Orco (**C**), and Orco homotetramer (**D**) in response to octanol and PA. Octanol was applied at a concentration of 30 μM for Or1a/Orco and 10 μM for the others, while PA was administered at 10 μM for all groups. Odorant-evoked currents were recorded using a two-electrode voltage-clamp technique. The Or/Orco-mediated currents activated by octanol or PA were reversible. All electrophysiological recordings were conducted at a holding potential of −80 mV (n = 5–7 from four different *Xenopus* oocytes).

To investigate the effect of PA on insect odorant receptors, we conducted two-electrode voltage-clamp electrophysiology using *Xenopus laevis* oocytes expressing *D. melanogaster* Ors. We tested the activation of Or1a, Or24a, and Or35a in combination with Orco. The application of PA induced inward currents in all three Or/Orco complexes in a concentration-dependent manner (Figure 2A–C). Control experiments confirmed that PA had no effect on uninjected oocytes, indicating a receptor-specific response. Or1a, Or24a, and Or35a were selected for this study based on their well-characterized expression and functional profiles in heterologous systems such as *Xenopus laevis* oocytes. These receptors have previously been reported to respond to esters and alcohols—chemical classes to which phenethyl acetate belongs—making them appropriate models for evaluating PA’s activation potential. Their inclusion also enabled us to assess whether PA activation is limited to specific odorant receptor subtypes or is broadly effective across receptors with diverse ligand selectivity.

To assess whether Orco alone could be activated by PA, we expressed Orco homomers in *Xenopus* oocytes. Notably, PA also induced inward currents in Orco homomeric receptors, whereas octanol, a known *D. melanogaster* Or ligand, failed to activate Orco (Figure 2D). This suggests that PA interacts directly with Orco, independent of specific Or subunits. To clarify the ligand specificity, we also evaluated the effect of octanol, a well-characterized *D. melanogaster* odorant, on the same receptor combinations. Octanol induced robust inward currents when applied to Or1a/Orco, Or24a/Orco, and Or35a/Orco complexes, confirming its known agonistic activity toward specific Ors. However, no response was observed in oocytes expressing Orco alone, indicating that octanol requires a functional Or subunit for receptor activation. In contrast, PA elicited responses in both Or/Orco heteromers and Orco homomers, suggesting a direct interaction with the Orco subunit. These results highlight the distinct receptor activation profiles of PA and octanol and emphasize the unique role of PA as a direct Orco agonist.

### 2.2. PA Activation Is Concentration-Dependent and Voltage-Independent

**Figure 3:**
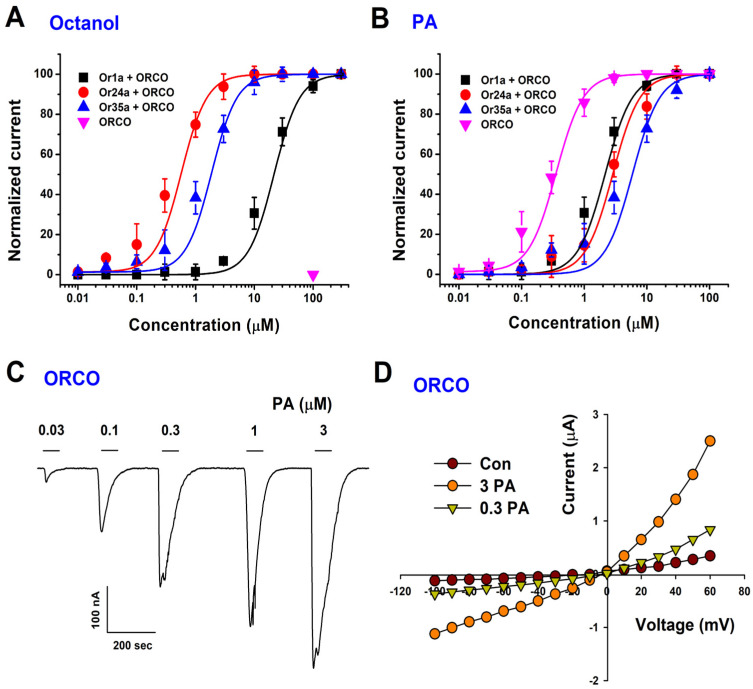
Concentration–response relationship of octanol or PA on Or1a, Or24a, or Or35a co-expressed with Orco, and current–voltage relationship of PA on Orco homotetramer. (**A**,**B**) Concentration–response curves of octanol or phenethyl acetate (PA) were generated for each Or/Orco complex. The PA activation curve for Or/Orco was fitted to the Hill equation. Data points represent the mean ± SEM. (**C**) The representative current trace illustrates the progressive activation of the Orco homotetramer by PA in a concentration-dependent manner. Electrophysiological recordings were performed at room temperature using a two-electrode voltage-clamp technique, with a holding potential of −80 mV. (**D**) Voltage-dependent properties of the Orco homomeric receptor were assessed by applying voltage ramps using the two-electrode voltage clamp. The membrane potential varied from 100 mV to +60 mV, with a holding potential of −80 mV. PA was applied at concentrations of 0.3 µM and 3 µM. Con indicates the control condition, representing the baseline current recorded from Orco-expressing oocytes without any odorant stimulation. Data were collected from 6 to 8 oocytes obtained from four different *Xenopus* frogs.

We next determined the concentration–response relationship of PA using TEVC recordings. PA activated Or1a/Orco, Or24a/Orco, Or35a/Orco, and Orco homomers with half-maximal effective concentrations (EC_50_) of 2.1 ± 1.2 μM, 2.8 ± 0.2 μM, 5.9 ± 1.1 μM, and 0.4 ± 0.2 μM, respectively (Figure 3A–C). Notably, Orco homomers exhibited the highest sensitivity to PA, suggesting a direct interaction between PA and Orco.

To examine the voltage dependence of PA activation, we performed current–voltage (I–V) measurements under varying membrane potentials. PA-induced currents exhibited a linear I–V relationship, indicating the voltage-independent activation of Orco (Figure 3D). These findings confirm that PA activates Orco through a mechanism distinct from classical voltage-gated channels.

### 2.3. Molecular Docking Identifies PA-Binding Site in Orco

**Figure 4:**
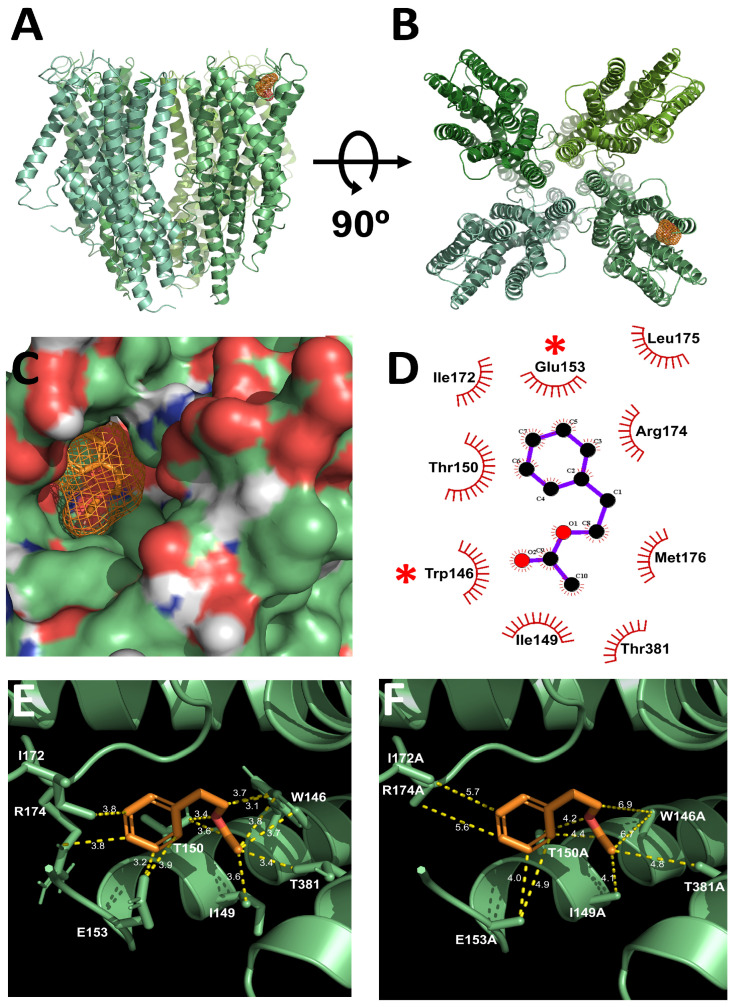
Validation of PA binding to the Orco homomeric receptor via molecular docking analysis. (**A**) Front-view representation of the binding interaction between PA and the Orco homomeric receptor. (**B**) Top-view representation of the PA–Orco interaction, rotated 90° relative to the front view. (**C**) Conformational binding interaction of PA with the wild-type Orco subunit. (**D**) Structural representation of the chemical interactions between PA and key residues of the Orco subunit. W146 and E153 residues, which were experimentally validated through site-directed mutagenesis, are indicated with asterisks (*) to distinguish them from other surrounding residues involved in ligand binding. (**E**) Visualization of PA binding to the wild-type Orco subunit, highlighting interaction distances and participating residues. (**F**) Comparison of PA binding to the mutant Orco subunit, illustrating changes in interaction distances and residue engagement. This figure provides a comprehensive visualization of PA–Orco interactions, detailing key binding residues and interaction distances.

**Figure 5:**
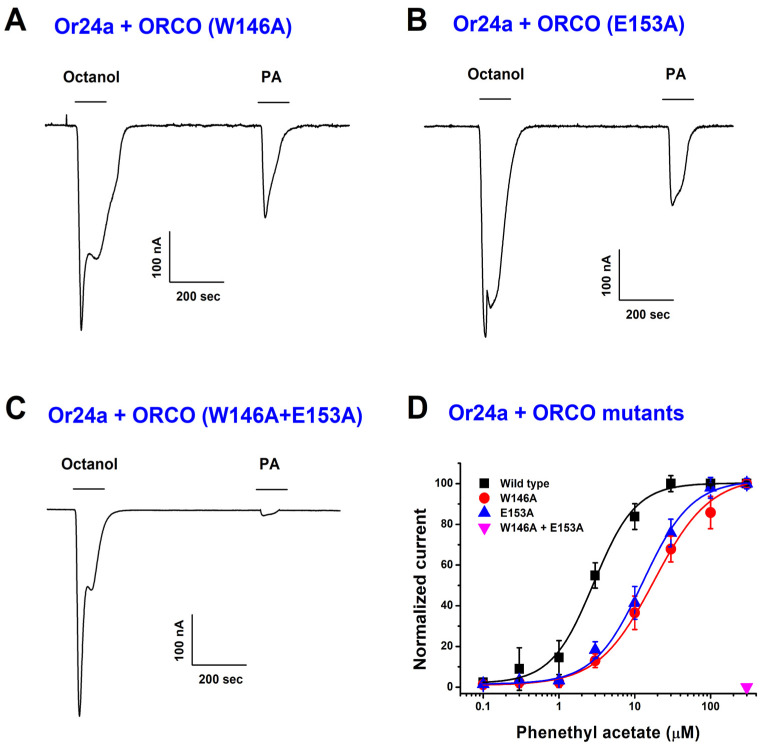
Verification of odorant-evoked inward currents in wild-type Or24a and mutant Orco subunit odorant receptors. (**A**–**C**) Inward currents induced by 10 µM of octanol and 10 µM of PA were examined in each receptor variant. Electrophysiological recordings were performed using a two-electrode voltage-clamp protocol, with a holding potential of −80 mV (n = 6–8, obtained from four different *Xenopus* oocytes). (**D**) Concentration–response curve for PA in wild-type Or24a/Orco and its mutant variants. Data were analyzed using the Hill equation for curve fitting.

To further investigate the molecular interaction between PA and Orco, we conducted molecular docking simulations. The docking model revealed that PA binds within a highly conserved region of the Orco subunit, specifically near W146 and E153 (Figure 4A–C). These residues are positioned within the extracellular domain, suggesting a potential ligand-binding site. To validate the predicted binding site, we generated W146A and E153A point mutations in Orco and examined their effects on PA activation. TEVC recordings showed that single mutations (W146A or E153A) significantly reduced PA-induced currents, while the double mutation (W146A + E153A) nearly abolished PA activation (Figure 5). These results confirm that W146 and E153 are critical residues for PA binding and receptor activation.

### 2.4. PA-Induced Activation Is Reduced in Orco Mutants

To further verify the functional significance of W146 and E153, we compared the EC_50_ values of PA on wild-type and mutant Orco receptors. The EC_50_ values for wild-type, W146A, and E153A Orco homomers were 0.4 ± 0.2 μM, 17.9 ± 2.1 μM, and 12.8 ± 1.9 μM, respectively, indicating a drastic reduction in PA sensitivity in mutant receptors (Figure 5A–D). These findings provide strong molecular evidence that PA binds directly to Orco and functions as an Orco agonist, opening new possibilities for its application in insect olfactory modulation. Electrophysiological recordings confirmed that the activation of Or1a, Or24a, and Or35a by octanol remained consistent when co-expressed with mutant Orco, indicating that the introduced mutations did not disrupt overall receptor functionality. This correlation between our docking model and patch-clamp results supports the conclusion that W146 and E153 are specifically involved in PA binding and activation, rather than affecting general Or/Orco function.

## 3. Discussion

Orco, a highly conserved co-receptor, has across the lineages conserved sequence [12,19,20]. Through topology and entropy value analysis, it was confirmed that the C-terminal region of the insect Orco protein is a highly conserved site [21,22,23]. Orco genes have been conserved for 250 million years, which has enabled the primary function, extracellular signal transduction to CNS, and heterodimerization with various Or proteins [22]. In the case of the C-terminal region, which has been preserved for each lineage, it is difficult to variably evolve according to the species-specific Or protein [24,25]. In the case of the N-terminal region, it is possible to undergo continuous evolution according to the lineage-specific Or protein [26,27]. The reason that different reactions can occur for each insect, even in the same odorant, is that the N-terminal of the Orco protein has been performing the role of a template that evolves according to the Or protein to be assembled over time. It was confirmed that the Orco subunit can be replaced with the Or subunit [19]. Insect odorant-specific responses are primarily mediated by the Or subunit, which determines ligand specificity. The Orco subunit, in contrast, is highly conserved across insect lineages and functions as a structural and functional co-receptor. Together, the Or and Orco form a heteromeric complex in which the variable Or subunit has evolved to detect ecologically relevant odorants specific to the insect’s habitat, while Orco provides the core ion channel functionality. It was confirmed that by using Consurf protein evolutionary data [28,29,30], the Orco protein pore part and the inter-subunit contact residue were highly conserved. The conserved region was closer to the C-terminal region pore in the 3D structure of the Orco homotetramer. It seems that the conserved sequences have been preserved across insect lineages, as they are essential for the basic functions: signal transduction, ion flux, and heterodimerization with various Or proteins.

Understanding how insect odorant receptors interact with volatile compounds is essential for advancing research in chemosensory biology and developing innovative pest management strategies. In this study, we demonstrate that PA functions as an agonist for the Orco receptor in *D. melanogaster*, triggering receptor activation in a concentration-dependent, reversible, and voltage-independent manner. By elucidating the molecular mechanisms underlying PA–Orco interactions, our findings contribute to a deeper understanding of insect olfactory signaling and provide a foundation for potential applications in insect behavior modulation. Our electrophysiological experiments in *Xenopus laevis* oocytes reveal that PA is capable of activating Or1a, Or24a, and Or35a when co-expressed with Orco, confirming its role as a ligand for insect Ors.

A particularly intriguing finding is that PA also activates Orco homomers, whereas octanol, a well-characterized *D. melanogaster* Or ligand, does not. This suggests that PA directly interacts with Orco, independent of specific Or subunits. Given that Orco is a highly conserved co-receptor across insect species, this discovery has broader implications beyond *D. melanogaster*, highlighting the potential for PA or similar compounds to serve as the universal modulators of insect olfactory function.

Our study demonstrates that PA functions as an Orco agonist, providing insights into the molecular mechanisms of insect olfactory receptor activation. Given that Orco is highly conserved across insect species, our findings suggest that PA or structurally related compounds could be explored as potential modulators of insect behavior. This could have practical applications in pest management strategies, such as developing attractants for monitoring and trapping target insect populations or designing compounds that disrupt olfactory-driven behaviors like host-seeking and mating. Furthermore, our research provides a foundation for future chemosensory studies by identifying key binding residues (W146 and E153) in Orco, which could facilitate the development of selective agonists or antagonists for insect olfactory modulation. These insights could aid in rational insect control strategies that minimize the environmental impact compared to traditional insecticides.

In this study, we confirmed the activation of PA on the Orco homotetramer receptor. Through in vitro experiments using TEVC, it was confirmed that PA acted as a ligand on the *D. melanogaster* Orco homotetramer receptor. PA is one of the aromatic compounds, and it is biosynthesized by the condensation of acetyl-CoA and phenethyl alcohol by the catalysis of alcohol acetyltransferase [31]. PA activation as a ligand on Orco homotetramer receptor was concentration-dependent, reversible, and voltage-independent. When the PA was applied on the Orco homotetramer with an appropriate concentration, it was confirmed that the PA-binding site existed in the Orco subunit. Through the interaction analysis between the 3D structure and the point-mutated Orco homotetramer protein, the W146 and E153 residues are the proposed PA-binding sites. Because when these residues were exchanged for alanines, that change did not affect the binding of positive control molecules but did affect the binding of PA. By analyzing the Orco protein structure, W146 is located in three segments and is adjacent to the lipid bilayer, and E153 is located in six segments and is located on the extracellular side of the C-terminal side [32]. The two positions are adjacent to each other in the folded protein structure, and the binding site of PA is close to the conserved region. The Orco protein sequence of this conserved region means it is possible that other insect lineages also have an Or activation effect by PA. As a result, PA may act as ligands not only for *D. melanogaster* Ors but also for other insect lineages Ors. At the molecular level, Or studies provide effective means for chemosensory studies to control only target insects. The research on the conserved region of insect Orco proteins can provide a good tool for the discovery of agents that can control pests across lineages. PA and similar volatile organic compounds provide a non-invasive and reversible means of influencing insect behavior. Additionally, compared to biological control agents, which can be costly and difficult to deploy in large-scale agricultural settings, PA-based strategies offer a scalable, cost-effective alternative. From an environmental perspective, PA is a naturally occurring compound derived from fermentation byproducts, making it a more sustainable option than conventional chemical insecticides, which often have negative effects on non-target organisms and contribute to pesticide resistance. By leveraging insect-specific olfactory pathways, PA and similar molecules could serve as species-selective control agents with minimal ecological disruption. The high sensitivity of the Orco receptor to PA (EC_50_ = 0.4 μM) suggests that insects may detect this compound at low concentrations under natural conditions. This level of responsiveness supports the feasibility of PA as a volatile attractant in semiochemical-based pest management tools, such as baited traps. However, further research is needed to evaluate whether PA elicits consistent behavioral attraction across insect species and ecological contexts. Field validation studies will be essential to determine its efficacy as a practical lure for monitoring or controlling pest populations.

This study provides molecular-level evidence that phenethyl acetate functions as an Orco agonist, activating insect Ors in a concentration-dependent manner. By integrating electrophysiological recordings, molecular docking simulations, and site-directed mutagenesis, we have identified key residues critical for PA binding and receptor activation. These findings not only advance our understanding of insect olfactory mechanisms but also open up new possibilities for developing species-specific, environmentally friendly strategies for insect control.

## 4. Materials and Methods

### 4.1. Materials

Phenethyl acetate, derived from acetic acid bacterial cultures, was selected as the model chemoattractant for this investigation. A variety of individual compounds were assessed to identify and select microbial metabolites with the most potent chemotactic properties. Phenethyl acetate, octanol, and all other chemicals used in this study were procured from Sigma-Aldrich (St. Louis, MO, USA). For the two-electrode voltage-clamp (TEVC) assay, all compounds were appropriately diluted with dimethyl sulfoxide (DMSO), ensuring that the final DMSO concentration remained below 0.01%.

### 4.2. In Vitro Transcription mRNA and Injection into Xenopus Oocyte

The *D. melanogaster* Or subunits (Or1a, Or24a, and Or35a) (FlyBase IDs: FBgn0029521, FBgn0026394, and FBgn0028946) and the odorant receptor co-receptor Orco (FlyBase ID: FBgn0037324) were synthesized as complementary DNA by Cosmogenetech (Seoul, Republic of Korea). The synthesized cDNA constructs were subcloned into the pGEM-HE expression vector. Plasmid transformation was performed using Escherichia coli DH5α competent cells, and recombinant plasmids were isolated using a miniprep protocol. The integrity of the cloned sequences was confirmed by Sanger sequencing. For complementary RNA synthesis, *D. melanogaster* Or1a, Or24a, Or35a, and Orco cDNA were linearized with appropriate restriction endonucleases, and digestion efficiency was verified via agarose gel electrophoresis. In vitro transcription was carried out using T7 RNA polymerase (mMESSAGE mMACHINE T7 Transcription Kit; Thermo Fisher Scientific, Waltham, MA, USA). The resulting mRNA was precipitated, resuspended in DEPC-treated water, and stored at −80 °C for subsequent experiments.

### 4.3. Xenopus Oocyte Preparation

*Xenopus laevis* frogs were obtained from the Korean *Xenopus* Resource Center for Research (KXRCR000001, Gangwon, Republic of Korea). The isolation and maintenance of *Xenopus* oocytes followed the standardized protocols described by Pyeon et al. [33] and complied with the ethical guidelines of Chonnam National University (CNU IACUC-YB-2016-07, July 2016). Microinjection of 50 ng of mRNA per oocyte was performed using a nanoliter injector (Drummond Scientific, Vernon Hills, IL, USA). Following injection, the oocytes were incubated at 18 °C in a shaking incubator to facilitate optimal protein expression before electrophysiological recordings.

### 4.4. Three-Dimensional Structure Modeling and Molecular Docking Studies

For the molecular docking study of the *D. melanogaster* Orco homotetramer, template protein structures were retrieved from the Protein Data Bank (PDB ID: 6C70, 3.50 Å resolution). The three-dimensional ligand structure of phenethyl acetate was obtained from PubChem (PubChem CID: 7654). Molecular docking simulations were performed using AutoDock Tools (version 4.2.6, La Jolla, CA, USA) from The Scripps Research Institute, incorporating parameters such as intermolecular binding energy, inhibition constant, crystallographic structures, and energy minimization. Docking of phenethyl acetate was executed using the default settings of AutoDock. The protein–ligand complex of phenethyl acetate and the Orco homotetramer was analyzed using LigPlot (version 4.5.3, EMBL-EBI, Hinxton, Cambridgeshire, UK) and PyMOL (version 1.8.4.2, Schrödinger, New York, NY, USA).

### 4.5. Data Recording with a Two-Electrode Voltage Clamp

The two-electrode voltage-clamp (TEVC) experiments were conducted following established protocols as described in Lee et al. [21]. This methodology ensures the accurate measurement of odorant receptor activation and has been widely used in previous studies on insect olfactory receptors. The molecular interaction between the odorant receptor (Or) and its ligand was examined using Or-expressing oocytes. Electrophysiological recordings were conducted with a two-electrode voltage-clamp system (OC-725C; Warner Instruments, Hamden, CT, USA) and digitized using a Digidata acquisition system (1322A; Molecular Devices, Sunnyvale, CA, USA). The oocyte membrane potential was clamped at −80 mV. Stock solutions of each compound were prepared by dissolving them in ND96 buffer (58.44 mM NaCl, 2 mM KCl, 1 mM MgCl_2_, 1.8 mM CaCl_2_, 5 mM HEPES, pH of 7.4) at the appropriate concentrations before use. Ligand application was performed at a perfusion rate of 2 mL/min. Ligand-induced inward currents were recorded and digitized using pClamp10 software (Axon Instruments, Union City, CA, USA).

### 4.6. Site-Directed Mutagenesis for Interaction Site Validation

To validate the potential binding sites predicted by 3D protein–ligand docking simulations, molecular characterization was conducted through site-directed mutagenesis. Point mutations were introduced into the insect odorant receptor using PCR-based mutagenesis. Custom primers were designed to modify specific amino acid residues, and mutant constructs were generated via PCR amplification. The amplified PCR products were transformed into XL-1 Blue supercompetent cells, and plasmid DNA was purified using a miniprep method. Sequence verification was performed by Gentech Inc. (Seoul, Republic of Korea). To confirm the binding site of PA, each mutant Orco subunit was co-expressed with the wild-type Ors receptor via microinjection. The effect of these mutations on PA-binding affinity was evaluated through two-electrode voltage-clamp recordings.

### 4.7. Data Statistics and Analysis

All values are presented as the mean ± S.E.M. (standard error of the mean) following data analysis. Data collected using the two-electrode voltage-clamp technique were analyzed with SigmaPlot 10.0 software. The fitted equation data were generated using OriginPro 9.0 software (Origin, MA, USA) and the Hill equation: y = V_min_ + (V_max_ + V_min_) * [x]^n^/([EC_50_]^n^ + [x]^n^), which represent the maximum and minimum currents, respectively, x is the concentration of the applied ligand, n is the Hill coefficient, and EC_50_ denotes the half-maximal effective concentration of PA. Statistical significance was set at *p*-values < 0.05.

## Data Availability

Raw sequences obtained by Nucleotide database have been made available through the NCBI Sequence Library. The datasets generated during and/or analyzed during the current study are available from the corresponding author on reasonable request.
